# Maturation-associated changes in Sertoli cells following *in vitro* culture of frozen-thawed prepubertal mouse testicular tissue

**DOI:** 10.3389/frph.2026.1835316

**Published:** 2026-04-23

**Authors:** Laura Moutard, Marion Dufour, Nathalie Rives, Magali Basille-Dugay, Eva Chemin, Valentin Rousseau, Aurélie Feraille, Christine Rondanino, Ludovic Dumont

**Affiliations:** 1 Inserm U1239-NorDiC—Team Adrenal and Gonadal Pathophysiology (AGoPath), University of Rouen Normandy, Rouen, France; 2 Institute for Research and Innovation in Biomedicine (IRIB), France; 3 Biology of Reproduction-CECOS Laboratory, Rouen University Hospital, Rouen, France

**Keywords:** cryopreservation, *in vitro* spermatogenesis, mouse, prepubertal testis, Sertoli cells

## Abstract

To preserve the fertility of prepubertal boys with cancer, testicular tissue can be cryopreserved in the hope of subsequently inducing their maturation after transplantation or *in vitro* culture for natural conception or medically assisted reproduction. Testicular tissue maturation procedures are currently at an experimental stage and mainly developed in animal models. Among these, *in vitro* sperm yield production in cultures of mouse prepubertal testicular explants is relatively low. Despite the essential role of Sertoli cells in the progression of spermatogenesis, the maturation and functionality of these somatic cells under organotypic culture have never been thoroughly studied. This work aims to investigate Sertoli cell maturation and functionality after *in vitro* maturation of frozen-thawed mouse prepubertal testicular. Frozen-thawed testicular fragments from 6-day-old prepubertal mouse tissues were cultured for 16 days (D16) and 30 days (D30). Immunohistochemical, RT-qPCR analyses as well as measurements of intratesticular AMH and inhibin B levels by ELISA were performed on cultured explants and on *postpartum* (d*pp*) *in vivo* controls. The number of Sertoli cells was higher at D30 than at 36 d*pp*. In addition, the expression of immature and mature Sertoli cell markers were similar between *in vitro* cultured explants and *in vivo* control tissues. Moreover, inhibin B, a marker of Sertoli cell functionality, was detected in cultured explants at D16 and D30. However, supplementation of culture media with 5 ng/mL of FSH, a pituitary hormone that regulates Sertoli cell functions, had little impact on the progression of the first wave of spermatogenesis from cultured prepubertal explants.

## Introduction

To preserve the fertility of prepubertal boys with cancer prior to highly gonadotoxic treatment, testicular tissue can be cryopreserved for subsequent maturation. Testicular tissue maturation, with the aim of producing spermatozoa for assisted reproductive technologies (ART), could be envisaged using *in vitro* or *in vivo* approaches but has not been clinically validated in humans to date. *In vitro* maturation approaches are currently being developed in animal models. *In vitro* spermatogenesis could indeed be later proposed to patients for whom testicular autografting is not indicated (testicular localization of residual malignant cells). Organotypic culture is the only *in vitro* maturation approach that preserves tissue architecture, testicular microenvironment and cellular interactions. It has been used to obtain spermatozoa from fresh or frozen-thawed mouse prepubertal testicular tissues (1–3). Oocyte microinjection with *in vitro* produced spermatozoa produced from fresh (1) and cryopreserved (4) prepubertal testicular tissues yielded viable and fertile mice. However, *in vitro* sperm production in the mouse model remains a rare event.

We have previously shown that the maturation and steroidogenic activity of Leydig cells, as well as androgen and estrogen signaling, are impaired in cultures of fresh and frozen-thawed prepubertal mouse testicular tissues (5). These defects may contribute to the low efficiency of *in vitro* spermatogenesis. In addition to Leydig cells, Sertoli cells also play an essential role in the progression of spermatogenesis. Indeed, Sertoli cells contribute to spermatogenic cell support, protection and nutrition (6), play a role in the paracrine and endocrine control of spermatogenesis, regulate cholesterol metabolism during spermatogenesis (7), participate in sperm movement by secreting fluids (8), phagocytose apoptotic cells and foreign bodies (9), secrete the Sertolian peptide hormone inhibin B (INHB), which suppresses follicle-stimulating hormone (FSH) release (10), and enable blood-testis barrier (BTB) formation. Surprisingly, the maturity and functionality of Sertoli cells in organotypic cultures, compared to *in vivo* condition, have never been examined in depth. Exploring the molecular profiles of different testicular cell populations in *in vitro*-matured explants, particularly Sertoli cells, may help identify mechanisms that limit the progression of *in vitro* spermatogenesis.

Sertoli cells are the first cells to differentiate in the undifferentiated fetal gonad, an event that enables the formation of seminiferous cords, the prevention of meiotic entry and the differentiation and function of Leydig cells (11). During puberty, the transition from immature to adult Sertoli cells takes place, and the role of these somatic cells shifts from being the organizing center of the gonad to supporting spermatogenesis by nourishing developing sperm cells throughout the stages of spermatogenesis.

The number of Sertoli cells is known to determine testicular size, the number of germ cells per testis and sperm production (12). Immature Sertoli cells proliferate up to 17 days *postpartum* (d*pp*) in mice (13), so that the final number of Sertoli cells is determined before adulthood. The proliferation and maturation of Sertoli cells are regulated by several factors, including the FSH, which acts via the FSH receptor (FSHR) expressed specifically in these cells (14). The effect of FSH varies according to the state of maturation of Sertoli cells (11). FSH regulates Sertoli cell proliferation during fetal and early postnatal life, whereas it regulates their differentiation after cessation of mitosis at puberty (15). In addition to FSH, the insulin/IGF signaling pathway is involved in the regulation of Sertoli cell proliferation. In mice lacking both the insulin receptor (*Insr*) and the IGF1 receptor (*Igf1r*) specifically in Sertoli cells (SC-*Insr*;*Igf1r*), testis size and sperm production were decreased at adulthood as a result of a reduced proliferation rate of Sertoli cells during the late fetal and early neonatal period (16). In addition, FSH has been shown to stimulate IGF1 expression and inhibit IGF-binding protein 3 (IGFBP3) expression in these cells (17, 18).

With puberty, the expression of anti-Müllerian hormone (*Amh*), one of the first genes to be activated in Sertoli cells starting from fetal life (19), is sharply reduced. The decreased secretion of AMH into the bloodstream coincides with the appearance of meiotic germ cells, the final maturation and the androgen sensitivity of Sertoli cells (20, 21). The main factors involved in the arrest of Sertoli cell proliferation and in their maturation are androgens, all-*trans* retinoic acid (a*t*RA) and estrogens. The androgen receptor (AR) is absent in fetal Sertoli cells and its expression becomes progressively stronger during mouse postnatal development (from 5 to 7 d*pp* and established by 14 d*pp*) (20, 22). In addition, the development of a transgenic model that prematurely expresses AR specifically in Sertoli cells (TgSCAR) demonstrated that androgens, by acting specifically via AR in Sertoli cells, induce their maturation (23). Moreover, the expression of CYP26B1, an enzyme that degrades a*t*RA, decreases in Sertoli cells towards puberty onset in an androgen-independent manner to allow entry into meiosis (24). In the presence of a*t*RA, a decrease in FSH-stimulated bromodeoxyuridine incorporation as well as an increase in the expression of cyclin-dependent kinase (CDK) inhibitor 1B (P27KIP1), a protein involved in cell cycle arrest and cell differentiation, have been observed in cultures of immature Sertoli cells (25). The main source of estrogens are Sertoli cells in the immature testis and Leydig cells in adults (26, 27). Estradiol production by Sertoli cells is regulated by FSH through an increasing *Cyp19a1* expression (28). It has been reported that estrogens reduce the number of Sertoli cells during periods of proliferation (29). Depending on the isoform of the estrogen receptor through which they exert their effect, these hormones can regulate Sertoli cell proliferation or maturation: estrogens modulate Sertoli cell proliferation via estrogen receptor (ER) alpha (ERα), while cell cycle exit, and differentiation involve ER beta (ERβ).

The BTB is established at puberty. Tight junctions, crucial components of the BTB, form between adjacent Sertoli cells and divide the seminiferous epithelium into basal and apical compartments (30). Among tight junction proteins, claudin 3 (CLDN3) plays a role in governing the migration of preleptotene spermatocytes across the BTB (31). Tight junctions are linked to the actin cytoskeleton via cytosolic scaffolding proteins such as *Zonula occludens* 1 (ZO-1) (32). Besides the expression of BTB proteins, adult Sertoli cells also produce inhibins A and B. Inhibins are dimeric peptide hormones composed of an *α* subunit (encoded by *Inha*) and a β subunit (βA encoded by *Inhba* for inhibin A and βB by *Inhbb* for INHB). *In vivo*, inhibins regulate FSH secretion via a negative feedback mechanism on the pituitary gland. The production of INHB in the mature testis is a sign of Sertoli cell activity, and is stimulated by FSH (33, 34).

Transferrin (TRF), also produced by mature Sertoli cells, plays a role in regulating iron homeostasis and supporting spermatogenesis (35). TRF plays a role by binding to iron in the surrounding environment and transporting it to germ cells and other testicular cells that require it for metabolic activities. In germ cells, iron is needed to support cell division and maturation. Transferrin secretion is positively regulated by FSH, insulin, testosterone, and retinol (Rol) (35). In addition, mature Sertoli cells play an important role in regulating the self-renewal and differentiation of spermatogonial stem cells via paracrine mechanisms by producing factors such as the glial cell line-derived neurotrophic factor (GDNF) (36).

We have previously shown that the expression of the IGF-signaling pathway (*e*.*g*., *Igfbp3*) have been extensively deregulated in cultures of mouse prepubertal testicular explants (37). In addition, we showed that the expression and localization of key BTB components, including claudin 11 (CLDN11), ZO-1 and connexin 43 (CX43), were successfully established and maintained in cultures of frozen-thawed mouse prepubertal testicular explants (38). However, we have also reported a decrease in *Cldn3* expression, as well as increased BTB permeability and altered meiotic and post-meiotic progression in these organotypic cultures (38). Since Sertoli cells have never been thoroughly studied under these culture conditions, the aim of the present work was to investigate their number, state of maturation and function after *in vitro* maturation of frozen-thawed mouse prepubertal testicular tissue.

## Materials and methods

### Animals

#### Ethical approval

All the experimental procedures were approved by the Institutional Animal Care and Use Committee of University of Rouen Normandy under the licence/protocol number APAFiS #38239.

#### Mice and testis collection

CD-1 mice (Charles River Laboratories, L'Arbresle, France) were housed in a temperature-controlled room (22–23 °C) under a 12-h light/dark cycle. Prepubertal 6 d*pp* male mice were euthanized by decapitation and underwent a bilateral orchidectomy. Testes were transferred to Petri dishes containing *α*-MEM without phenol red (Gibco by Life Technologies, Saint-Aubin, France) and the complete removal of the tunica albuginea was performed with two needles under a binocular magnifier (S8AP0, Leica Microsystems GmbH, Wetzlar, Germany). Testes were then cultured after a freezing/thawing cycle (Supplementary Figure S1). Moreover, mice aged 22 and 36 d*pp* were euthanized by CO_2_ asphyxiation and their testes were used as the *in vivo* controls for 16 and 30 days of culture, respectively (Supplementary Figure S1). A total of 108 mice were used for this study with 4–8 biological replicates for each condition.

### Controlled slow freezing (CSF) and thawing procedure

Our team recently demonstrated that the impact of CSF had little (if any) impact on testicular explants protein (39) or transcriptome (37) expression, compared with fresh *in vitro* culture explants. For this reason, and in order to approximate as closely as possible the physiological conditions found in the clinic, the analyses performed for *in vitro* maturation were carried out using only frozen prepubertal testicular tissues.

#### CSF procedure

Testes were placed into cryovials (Dominique Dutscher, Brumath, France) containing 1.3 mL of the following cryoprotective medium: Leibovitz L15 medium (Eurobio, Courtabœuf, France) supplemented with 1.5 M dimethylsulfoxide (DMSO, Sigma-Aldrich, Saint-Quentin Fallavier, France), 0.05 M sucrose (Sigma-Aldrich), 10% (v/v) fetal calf serum (FCS, Life Technologies) (40) and 3.4 mM vitamin E (Sigma-Aldrich). After a 30 min equilibration at 4 °C, samples were frozen in a programmable freezer (Nano Digitcool, CryoBioSystem, L'Aigle, France) with a CSF protocol: start at 5 °C, then −2 °C/min until reaching −9 °C, stabilization at −9 °C for 7 min, then −0.3 °C/min until −40 °C and −10 °C/min down to −140 °C. Testicular tissues were then plunged and stored in liquid nitrogen and stored for >72 h and up to several months.

#### Thawing procedure

Cryotubes were warmed for 1 min at room temperature (RT) and then for 3 min in a water bath at 30 °C. They were then successively incubated at 4 °C in solutions containing decreasing concentrations of cryoprotectants for 5 min each [solution 1: 1 M DMSO, 0.05 M sucrose, 10% FCS, 3.4 mM vitamin E, Leibovitz L15; solution 2: 0.5 M DMSO, 0.05 M sucrose, 10% FCS, 3.4 mM vitamin E, Leibovitz L15; solution 3: 0.05 M sucrose, 10% FCS, 3.4 mM vitamin E, Leibovitz L15; solution 4: 10% FCS, 3.4 mM vitamin E, Leibovitz L15].

### Organotypic culture

*In vitro* cultures explants were performed as previously described (1, 2). Briefly, prepubertal 6-day old mouse testes, which contain spermatogonia as the most advanced type of germ cells, were first cut into four fragments (approximately 0.75 mm^3^ each, which was previously determined to be the most appropriate size for mouse *in vitro* spermatogenesis) (41). Fragments were placed on top of two 1.5% (w/v) agarose Type I gels (Sigma-Aldrich) soaked in culture medium since the day before and half-soaked in medium the day of the culture. Testicular fragments were then cultured under 5% CO_2_ at 34 °C for 16 days (D16), which corresponds to the end of meiosis and the appearance of the first-round spermatids, or 30 days (D30) to explore the end of the first spermatogenic wave. The basal medium (BM) contained *α*-MEM without phenol red, 10% KSR (KnockOut Serum Replacement, Gibco by Life Technologies), 0.1 mg/mL streptomycin and 100 IU/mL penicillin (Sigma-Aldrich). Rol (1 µM, Sigma-Aldrich) was added in all organotypic cultures from D2 and then every 8 days to respect the cycle of meiotic initiation (2, 41). Furthermore, the BM was supplemented or not with 5 ng/mL FSH (Biotechne, Noyal-Chatillon-sur-Seiche, France) from D10 to assess the functionality of Sertoli cells (Supplementary Figure S1). Media were prepared just before use and were replaced twice a week.

### Immunohistological analyses

#### Tissue fixation, processing and sectioning

Testicular tissues and fragments were fixed with Bouin's solution (Sigma-Aldrich) for immunohistochemical staining or 4% paraformaldehyde (Sigma-Aldrich) for immunofluorescence (2 h at RT for 6 d*pp* testes and cultured explants, overnight at RT for 22 and 36 d*pp* testes). They were then dehydrated in ethanol and embedded in paraffin. Tissue sections (3 μm thick) were prepared with the RM2125 RTS microtome (Leica) and were mounted on Polysine slides (Thermo Fisher Scientific, Waltham, MA, USA).

#### Immunohistochemical staining

For AMH, after deparaffinization, rehydration and antigen retrieval in 10 mM citrate buffer pH = 6.0 for 40 min at 96 °C, endogenous peroxidases were blocked with HP Block (Dako, Les Ulis, France) for 15 min and non-specific binding sites were blocked with Ultra-V Block solution (Thermo Scientific) for 5 min at RT. For CREM, after deparaffinization, tissue sections were heated in pH = 6.0 buffer (TA-999-DBHL, Agilent Technologies, Les Ulis, France), used as a 1:15 working solution (100 mL buffer + 1,400 mL Milli-Q® water), for 20 min at 95 °C in the PT Link device (Agilent Technologies). Slides were cooled at 65 °C and rinsed in phosphate buffered saline (PBS) for 5 min. Endogenous peroxidases were blocked with 3% H_2_O_2_ for 10 min. Non-specific sites were blocked with 5% (w/v) bovine serum albumin (BSA, Sigma-Aldrich) and 20% (v/v) goat serum (Sigma-Aldrich). Tissue sections were then incubated overnight at 4 °C with anti-AMH [1:200, MCA2246T, clone (5/6), Biorad, Marnes-la-Coquette, France] or anti-CREM [1:50, sc-440, clone (X-12) Santa Cruz Biotechnology, Heidelberg, Germany] antibodies. After three 5 min washes in PBS (for AMH) or PBS with 0.05% Tween-20 (PBST) (for CREM), slides were incubated for 10 min at RT with biotinylated polyvalent secondary antibody (UltraVision Detection System HRP kit, Thermo Scientific). After three 5 min washes in PBS (for AMH) or PBST (for CREM), a 10 min incubation at RT with streptavidin-peroxidase conjugate (UltraVision Detection System HRP kit, Thermo Scientific) was performed. The labeling was revealed after application of a chromogenic substrate (3,3′-diaminobenzidine tetrahydrochloride, Thermo Scientific) for 2 min (for AMH) or 1 min (for CREM) at RT. Images were acquired on an Axioscope 7 microscope (Zeiss, Rueil Malmaison, France) at a ×400 magnification. In 30 cross-sectioned seminiferous tubules (ST) per sample, we determined the proportion of CREM-positive ST.

#### Immunofluorescence staining

After deparaffinization, tissue sections were heated in citrate buffer pH = 6.0 (Agilent Technologies) for 30 min at 95 °C in the PT Link device (Agilent Technologies). Slides were cooled at 65 °C and rinsed in PBS for 5 min. Non-specific sites were blocked with 5% (w/v) BSA and 5% (v/v) horse serum (Sigma-Aldrich). Tissue sections were then incubated overnight at 4 °C in a humidified chamber with primary antibody {1:100, AR, ab133273, clone [EPR1535(2)], Abcam; 1:200, SOX9, ab5535, Merck Millipore, Darmstadt, Germany}, rinsed 3 times in PBST and incubated 1 h at RT with appropriate secondary antibody [1:200, Alexa 594-conjugated goat anti-rabbit, ab150080, clone (2B10), Abcam, Paris, France]. Sections were washed, dehydrated with ethanol, and mounted in Vectashield with Hoechst. Images were acquired on a THUNDER Imager 3D Tissue microscope (Leica) at a ×400 magnification. In 30 cross-sectioned ST per sample, we determined the proportion of positive ST (100% with ≥1 Sertoli-positive cell; data not shown), the percentage of Sertoli cells per ST (relative to germ cells), the number of Sertoli-positive cells per 1,000 µm² of ST (Sertoli-cell density), and the germ-to-Sertoli cell ratio.

### RNA extraction and RT-qPCR

#### RNA extraction

Total RNA was extracted from testicular samples using RNeasy Micro kit (Qiagen, Courtabœuf, France) according to the manufacturer's instructions. For *in vitro* cultured explants, we systematically carefully excised necrotic areas before RNA extraction to avoid contaminating RT-qPCR readouts with apoptosis transcripts, so that transcript levels in the healthy part of the samples (*i*.*e*., where *in vitro* spermatogenesis occurs) could be measured and compared with *in vivo* controls. Necrosis was identified visually under a stereomicroscope (*i*.*e*., dull and opaque grey-brown aspect, loss of translucency or elasticity, friability). To avoid contamination with genomic DNA, extracted RNA was incubated with two units of TURBO DNase (Life Technologies) for 45 min at 37 °C. The amount of RNA was measured with a NanoDrop spectrophotometer (NanoDrop Technologies, Wilmington, DE, USA) and its purity was determined by calculating the ratio of optical densities at 260 nm and 280 nm. RNA samples were purified using the Monarch RNA Cleanup kit (New England Biolabs, Évry-Courcouronnes, France).

#### Reverse transcription

The reverse transcription reaction was performed from 1 µg total RNA, using the MMLV-RT kit (Promega, Charbonnières-les-Bains, France) containing 200 U of MMLV-RT, 25 U of RNAsin, 0.5 µg of random primers, 5 µg of 5X buffer and 10 mM deoxyribonucleotides, in a final volume of 25 µg for 1 h at 37 °C. The complementary DNA (cDNA) samples obtained were diluted at 1:10.

#### Polymerase chain reaction

cDNA amplifications were carried out in a total volume of 20 μL containing 2.5 µL of cDNA, 10 μL of Luna Universal qPCR Master Mix (New England Biolabs) and 300 nM of each primer. Specific primers are listed in Supplementary Table S1. Reactions were performed in 96-well plates in a StepOnePlus thermocycler (Applied Biosystems, Thermo Fisher Scientific). The amplification condition was 20 s at 95 °C followed by 40 cycles (3 s at 95 °C, 30 s at the hybridization temperature: between 58 and 63 °C) and a final step of denaturation of 15 s at 95 °C, 1 min at 60 °C and 15 s at 95 °C. Melting curves were obtained to ensure the specificity of PCR amplifications. The size of the amplicons was verified by agarose gel electrophoresis (E-gel 4%, Life Technologies). The relative expression level of each gene was normalized to two housekeeping genes as recommended by the MIQE guidelines (42). RT-qPCR data were normalized to the geometric mean of *Gapdh* and *Actb*, previously identified as stable reference genes during mouse testis development (43). Throughout, “normalized to *Gapdh* and *Actb*” denotes normalization to their combined geometric-mean factor (not sequential normalization to two separate transcripts). For cell type-specific analyses, an additional scaling was applied: Sertoli-specific transcripts were further normalized to the geometric mean of *Sox9* and *Wt1* to account for variation in Sertoli-cell content, and Leydig-specific transcripts were further normalized to *Hsd3b* to account for Leydig-cell content. Relative expression was calculated using the 2^−*ΔΔ*Ct^ method (44).

### Enzyme-Linked immunosorbent assay (ELISA)

AMH levels were measured in 50 µL of testicular homogenates and in 50 µL of culture media using the mouse Anti-Mullerian Hormone ELISA kit (abx153628, Abbexa, Cambridge, UK), according to the supplier's recommendations. The sensitivity limit for the AMH assay was 0.05 ng/mL between 0.12 ng/mL and 10 ng/mL, with intra- and inter-trial variations of <10% and <12%, respectively. INHBB levels were measured in testicular homogenates and in culture media using the RayBio human/mouse/rat INHB Enzyme Immunoassay kit (EIA-INB, RayBiotech, Norcross, GA, USA), according to the supplier's recommendations. The sensitivity limit for the INHBB assay was 2 pg/mL between 1 pg/mL and 10,000 pg/mL, with intra- and inter-trial variations of <10% and <15%, respectively.

### Statistical analyses

Data are presented as means ± SEM. Statistical analyses were carried out with the R language^1^ (R Foundation for Statistical Computing, Vienna, Austria) and Rstudio software^2^ (RStudio, Inc., Boston, MA, USA). The distribution of data according to the normal distribution was verified using the Shapiro–Wilk test and the Jacque-Bera test, and the homogeneity of variances was verified using the Levene test. When the data followed a normal distribution with equal variance, the ANOVA test was used and then Tukey HSD *post hoc* test was used to compare the different groups. When the data followed a normal distribution with unequal variance, the ANOVA test with Welch's correction was used. Due to unequal variance and unequal sample size, we then used Dunnett T3 *post hoc* test to compare the different groups (45). When the data did not follow a normal distribution, the Kruskal–Wallis test was used. Due to unequal sample size, we then used Dunn *post hoc* test to compare the different groups. A value of *P* < 0.05 was considered statistically significant.

## Results

### Maintenance of Sertoli cells population throughout *in vitro* culture

SOX9 immunofluorescence was initially performed to detect and quantify Sertoli cells during mouse postnatal development and in *in vitro* cultured testicular tissues. The transcript levels of genes exclusively expressed in Sertoli cells (*Sox9*, *Wt1*) were then assessed by RT-qPCR.

**Figure 1:**
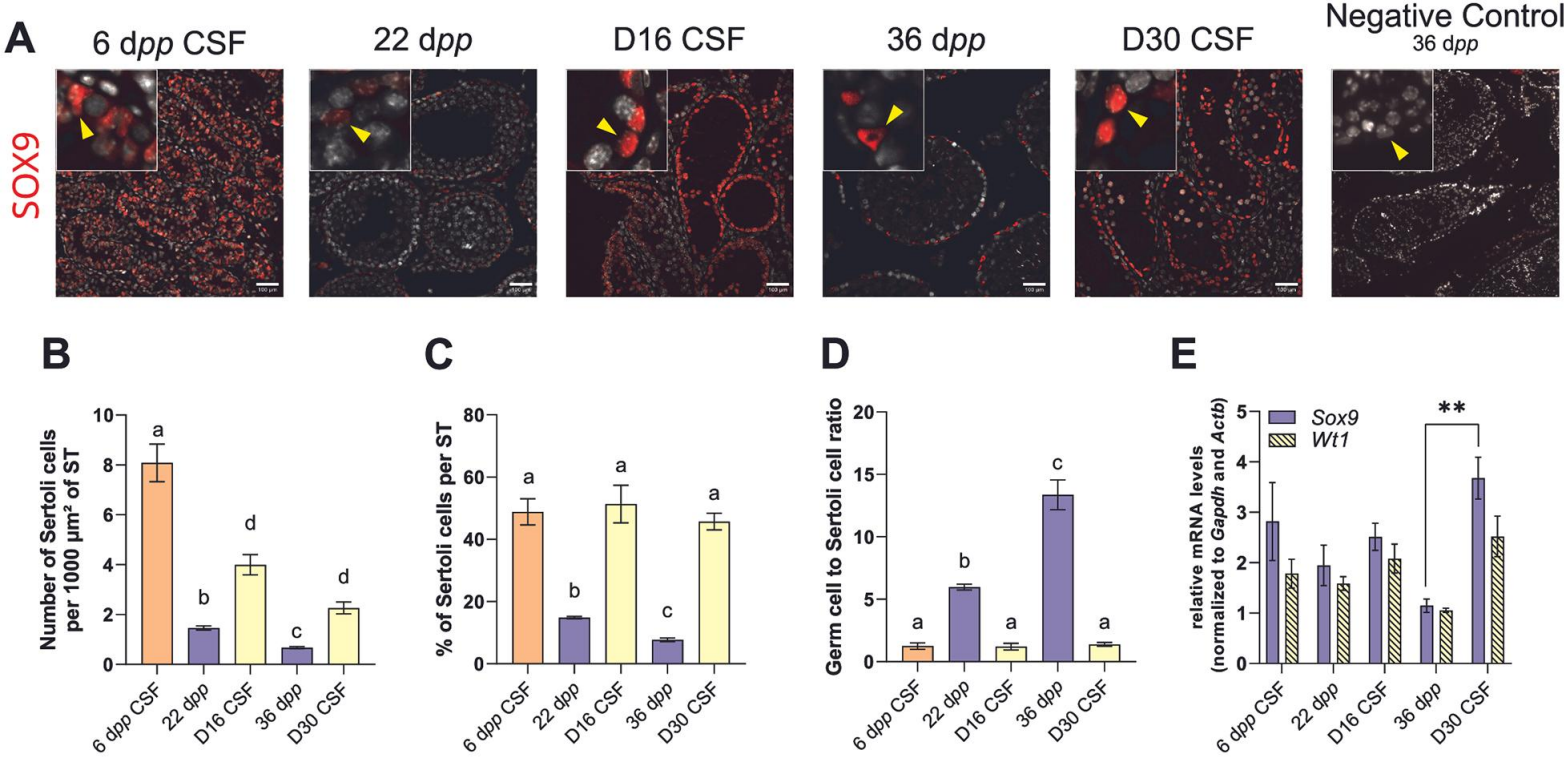
Sertoli cell content after 16 or 30 days of culture. **(A)** Representative images of SOX9 expression (in red) by Sertoli cells during mouse postnatal development (6, 22 and 36 d*pp*) and in *in vitro* cultures of frozen-thawed testicular tissues at D16 or D30. Testicular tissue sections were counterstained with Hoechst (in grey). Scale: 100 µm. Photomicrographs at a 5× additional digital magnification are shown. Yellow arrowheads indicate Sertoli cell. **(B)** Number of SOX9^+^ Sertoli cells per 1,000 µm² of seminiferous tubule (ST) *in vivo* (6, 22 and 36 d*pp*) and in *in vitro* cultured explants (D16 and D30). **(C)** Percentage of SOX9^+^ Sertoli cells per ST *in vivo* (6, 22, 36 d*pp*) and *in vitro* (D16 and D30). **(D)** Germ cell to Sertoli cell ratio *in vivo* (6, 22 and 36 d*pp*) and *in vitro* (D16 and D30). **(E)** Relative mRNA levels of Sertoli cell markers (*Sox9*, *Wt1*) (normalized to *Gapdh* and *Actb*). Data are presented as means ± SEM with *n* = 4-6 biological replicates for each group. Different letters denote significant differences between groups (*P* *<* 0.05). Identical letters between groups indicate that there is no significant difference between these groups (*P* ≥ 0.05). **P < 0.01. CSF, controlled slow freezing; D, days of culture; d*pp*, days *postpartum*; ST, seminiferous tubule.

The number of Sertoli cells per 1,000 µm² of seminiferous tubule (ST) remained lower in culture across maturation, partly mirroring—but less pronounced than—the *in vivo* pattern (Figure 1B). *In vivo*, the proportion of Sertoli cells per seminiferous tubule naturally declines at the expense of an expanding germ-cell population, while Sertoli cells exit mitosis around 16–17 d*pp*. Accordingly, in our *in vivo* controls, both the Sertoli-cell count per 1,000 µm² and the percentage of Sertoli per seminiferous tubule decreased from 6 to 36 d*pp*, with a reciprocal increase in the germ-to-Sertoli ratio (Figures 1A–D). In culture, by contrast, the percentage of Sertoli per seminiferous tubule and the germ-to-Sertoli ratio remained approximately at the 6 d*pp* (corresponding to D0 CSF) baseline throughout the culture period (Figures 1C,D), consistent with the limited germ-cell expansion observed *in vitro*.

Nevertheless, *Sox9* and *Wt1* mRNA levels were similar under *in vitro* and *in vivo* conditions, with the exception of an increase in *Sox9* transcript levels at D30 compared with 36 d*pp* (Figure 1E).

### Acquisition of maturation-associated molecular features by Sertoli cells throughout *in vitro* culture

The transcript levels of immature (*Amh*, *Krt18*) and maturation-associated (*p27Kip1*, *Ar*) Sertoli cell markers were measured by RT-qPCR during mouse postnatal development and in *in vitro* cultured testicular tissues. The expression of AMH and AR was analyzed after immunostaining. Moreover, the concentration of AMH was measured by ELISA in testicular homogenates and in organotypic culture media.

#### Assessment of immature Sertoli cells markers

**Figure 2:**
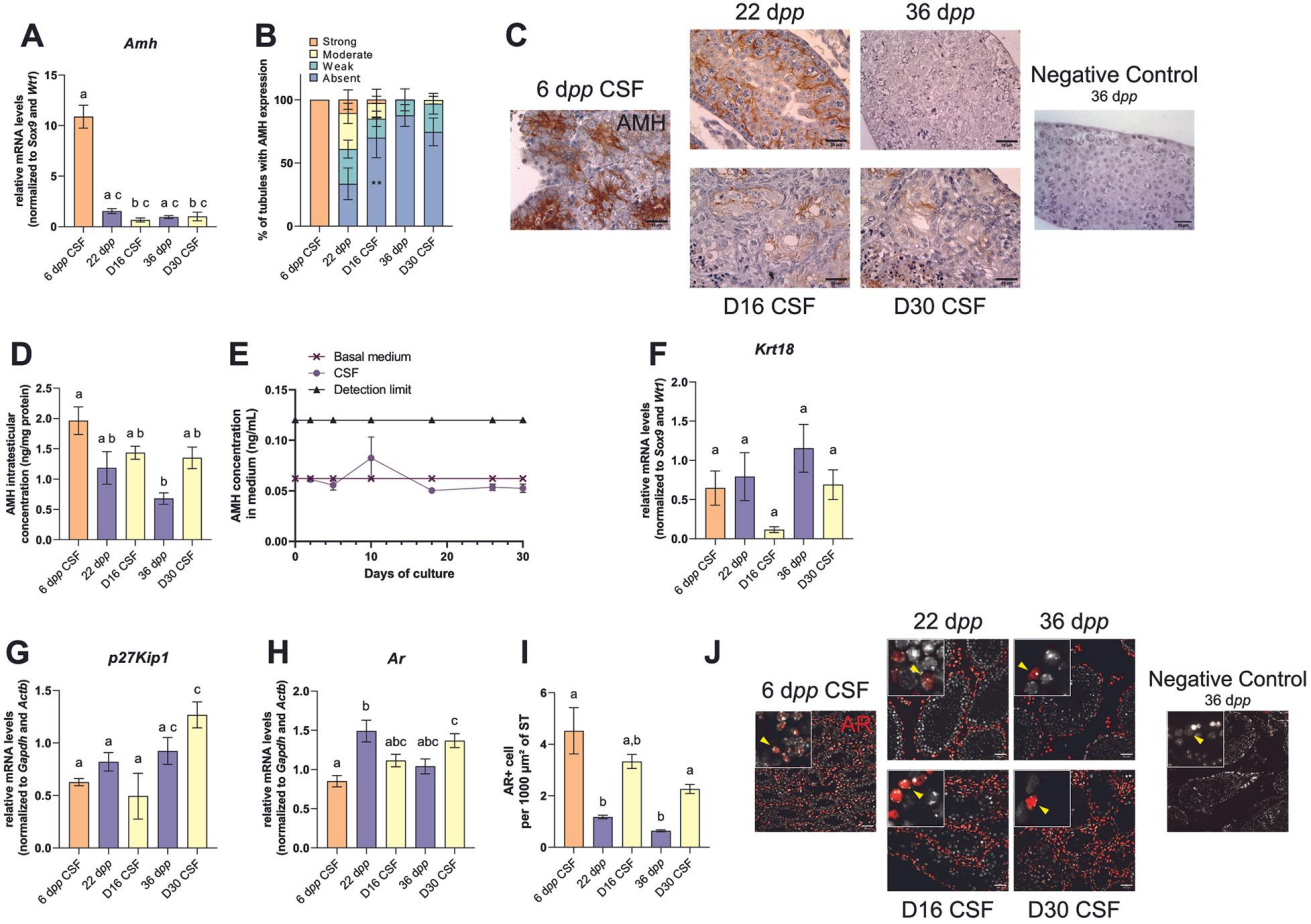
Sertoli cell maturation after organotypic culture. **(A)** Relative mRNA levels of *Amh* (immature Sertoli cell marker) normalized to *Sox9* and *Wt1* during mouse postnatal development (6, 22 and 36 d*pp*) and in *in vitro* cultured CSF explants after 16 days (D16) or 30 days (D30). **(B)** Mean percentage of tubules with AMH expression. AMH staining was assessed semi-quantitatively according to staining intensity: absent, weak, moderate or strong. **(C)** Representative images of AMH expression by Sertoli cells during mouse postnatal development and in *in vitro* cultured explants. Testicular tissue sections were counterstained with hematoxylin. Scale: 25 µm. AMH concentrations in **(D)** testicular tissues (ng/mg of protein) or **(E)** culture media (ng/mL). **(F–H)** Relative mRNA levels of immature (*Krt18*) or mature (*p27Kip1*, *Ar*) Sertoli cell markers (normalized to *Sox9* and *Wt1* or to *Gapdh* and *Actb*). **(I)** Number of AR^+^ cells per 1,000 µm² of seminiferous tubule *in vivo* (6, 22 and 36 d*pp*) and in *in vitro* cultured explants (D16 and D30). **(J)** Representative images of AR expression (in red) during mouse postnatal development (6, 22 and 36 d*pp*) and in *in vitro* cultures of frozen-thawed testicular tissues at D16 or D30. Testicular tissue sections were counterstained with Hoechst (in grey). Scale: 100 µm. Photomicrographs at a 5× additional digital magnification are shown. Yellow arrowheads indicate Sertoli cell. Data are presented as means ± SEM with *n* = 6 biological replicates for each group. Different letters denote significant differences between groups (*P* *<* 0.05). Identical letters between groups indicate that there is no significant difference between these groups (*P* ≥ 0.05). **P < 0.01. CSF, controlled slow freezing; D, days of culture; d*pp*, days *postpartum*; ST, seminiferous tubule.

*Amh* and *Krt18* mRNA levels were similar in *in vitro* matured tissues and *in vivo* age-matched controls at all time points (Figures 2A–F), consistent with a progressive reduction of immature Sertoli cell features over time in both conditions. A higher proportion of ST was observed to be free of AMH expression at D16 compared to 22 d*pp*; however, no significant difference was observed between D30 and 36 d*pp* (Figures 2B,C). In addition, intratesticular AMH concentrations were comparable after 16 or 30 days of culture compared to 22 or 36 d*pp* (Figure 2D). By contrast, AMH remained undetectable in the culture medium throughout the culture period (below the assay detection limit, Figure 2E).

#### Evaluation of markers associated with a mature Sertoli cell state

*p27Kip1* and *Ar* mRNA levels were similar in *in vitro* and *in vivo* matured tissues at all time points (Figures 2G,H) consistent with the acquisition of maturation-associated Sertoli cell features over time. In addition, the number of AR^+^ cells per 1,000 µm² of ST was higher at D30 than at 36 d*pp* but was not significantly different between D16 and 22 d*pp* (Figures 2I,J). This finding indicates the presence of AR-expressing Sertoli cells in cultured explants; however, AR immunostaining alone does not demonstrate restoration of normal AR-dependent Sertoli cell function.

### Several markers associated with Sertoli cell function are expressed in organotypic cultures

The transcript levels of several markers associated with Sertoli cell function were measured by RT-qPCR in organotypic cultures and *in vivo* controls. The concentrations of inhibin B (INHBB), a Sertolian peptide hormone, were also measured by ELISA in testicular homogenates and in organotypic culture media.

**Figure 3:**
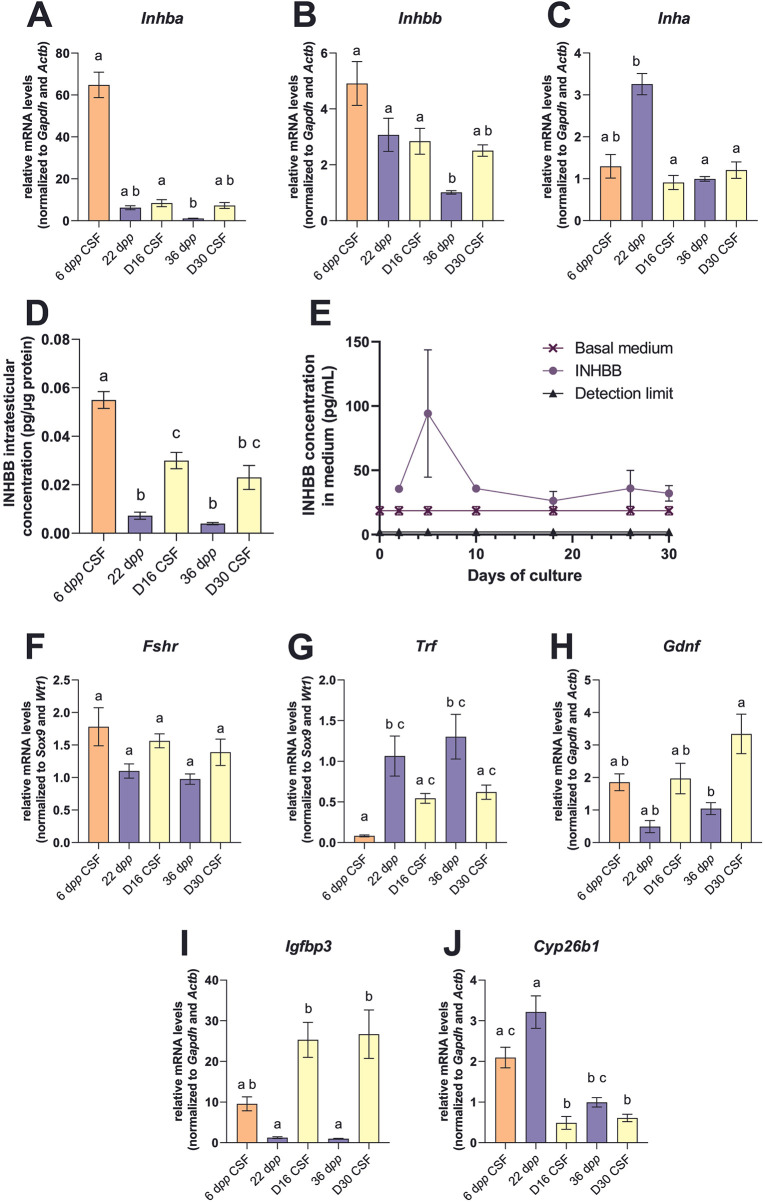
Sertoli cell functionality after organotypic culture. **(A–C)** Relative mRNA levels of *Inhba, Inhbb* and *Inha* normalized to *Gapdh* and *Actb* during mouse postnatal development (6, 22 and 36 d*pp*) and in *in vitro* cultured CSF tissues after 16 days (D16) or 30 days (D30). INHBB concentrations in **(D)** testicular tissues (pg/µg of protein) or **(E)** culture media (pg/mL). **(F–J)** Relative mRNA levels of *Fshr*, *Trf*, *Gdnf*, *Igfbp3* and *Cyp26b1* normalized to *Sox9* and *Wt1* or to *Gapdh* and *Actb*. Data are presented as means ± SEM with *n* = 6 biological replicates for each group. Different letters denote significant differences between groups (*P* *<* 0.05). Identical letters between groups indicate that there is no significant difference between these groups (*P* ≥ 0.05). CSF, controlled slow freezing; D, days of culture; d*pp*, days *postpartum*; INHBB, inhibin B; ST, seminiferous tubule.

The transcript levels of the gene encoding the *α* subunit of inhibin (*Inha*) was lower at D16 than at 22 d*pp* but was comparable between D30 and 36 d*pp* (Figure 3C). The mRNA levels of genes encoding the β subunit of inhibins (*Inhba*, *Inhbb*) were similar in cultured testicular explants and in *in vivo* tissue controls (Figures 3A,B). Intratesticular INHBB concentrations were significantly increased after 16 days of culture compared to 22 d*pp* but were not significantly different between D30 and 36 d*pp* (Figure 3D). INHBB was consistently detected in culture supernatants throughout the cultivation period (well above the detection limit), confirming a secretion into the medium, with a peak at the start of culture (Figure 3E).

The mRNA levels of *Fshr* and *Trf* were similar between *in vitro* and *in vivo* matured tissues at all time points (Figures 3F,G). The transcript levels of *Gdnf* were similar between D16 and 22 d*pp* but were higher at D30 than at 36 d*pp* (Figure 3H). Remarkably, *Igfbp3* transcript levels were significantly increased at D16 and D30 (25- to 27-fold) compared to their respective age-matched *in vivo* controls (Figure 3I). The mRNA levels of *Cyp26b1* were lower at D16 than at 22 d*pp* but were not significantly different between D30 and 36 d*pp* (Figure 3J).

### Supplementation with 5 ng/mL FSH from D10 has little impact on the culture of mouse prepubertal testicular explants

FSH is known to act through FSHR on Sertoli cells to regulate their proliferation, maturation-associated processes and function, as well as to stimulate spermatogenesis. Although we found that markers of Sertoli cell maturity and functionality were expressed in organotypic cultures even in the absence of FSH in the culture medium, we tested different FSH concentrations (1, 5 and 10 ng/mL) and analyzed their impact on *in vitro* spermatogenesis.

#### Impact on spermatogenesis

**Figure 4:**
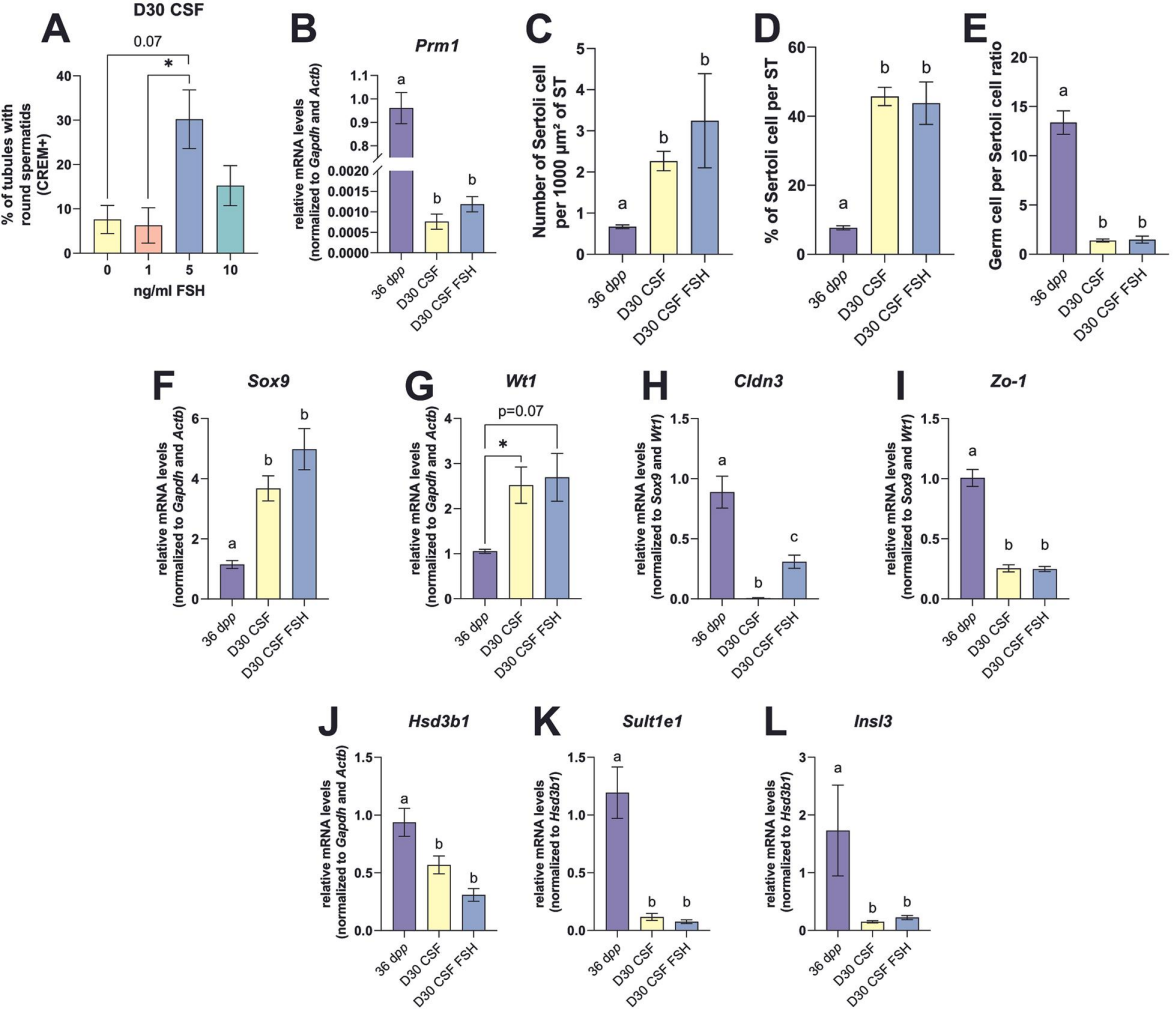
Effect of FSH on testicular tissues after 30 days of organotypic culture. **(A)** Different FSH concentrations (1, 5 or 10 ng/mL) were added to the culture media at D10 and the mean percentage of tubules with CREM^+^ round spermatids was determined at D30. **(B)** Relative mRNA levels of *Prm1* (spermatid marker) normalized to *Gapdh* and *Actb* at 36 d*pp* and in 30-day cultured CSF tissues with or without 5 ng/mL of FSH. **(C)** Number of SOX9^+^ Sertoli cells per 1,000 µm² of seminiferous tubule (ST) at 36 d*pp* and D30 ± FSH. **(D)** Percentage of SOX9^+^ Sertoli cells per ST. **(E)** Germ cell to Sertoli cell ratio. **(F–L)** Relative mRNA levels of Sertoli cell markers (*Sox9*, *Wt1*), blood-testis barrier markers (*Cldn3*, *Zo-1*), and relative mRNA levels of Leydig cell/steroidogenic markers (*Hsd3b*, *Slt1e1*, *Insl3*). Data are presented as means ± SEM with *n* = 4-8 biological replicates for each group. Different letters denote significant differences between groups (*P* *<* 0.05). Identical letters between groups indicate that there is no significant difference between these groups (*P* ≥ 0.05). *P < 0.05. CSF, controlled slow freezing; D, days of culture; d*pp*, days *postpartum*; ST, seminiferous tubule.

Since the highest percentage of ST containing at least one round spermatid was obtained at D30 when adding 5 ng/mL of FSH in the culture medium (Figure 4A and Supplementary Figure S2), this concentration was selected for subsequent analyses. However, even though this concentration of FSH in the culture medium led to a greater presence of round spermatids, mRNA levels of *Prm1* at D30 were drastically reduced compared with *in vivo* controls (Figure 4B).

#### Impact on the BTB

FSH supplementation had no impact on *Zo-1* transcript level (Figure 4I). Only *Cldn3* mRNA levels were significantly higher in cultures supplemented with FSH, although remaining significantly lower than at 36 d*pp* (Figure 4H).

#### Impact on Leydig cells

FSH supplementation had no impact on the gene expression of several Leydig cell markers (*i*.*e*., *Hsd3b1*, *Sult1e1*, *Insl3*) (Figures 4J–L).

#### Impact on Sertoli cells

**Figure 5:**
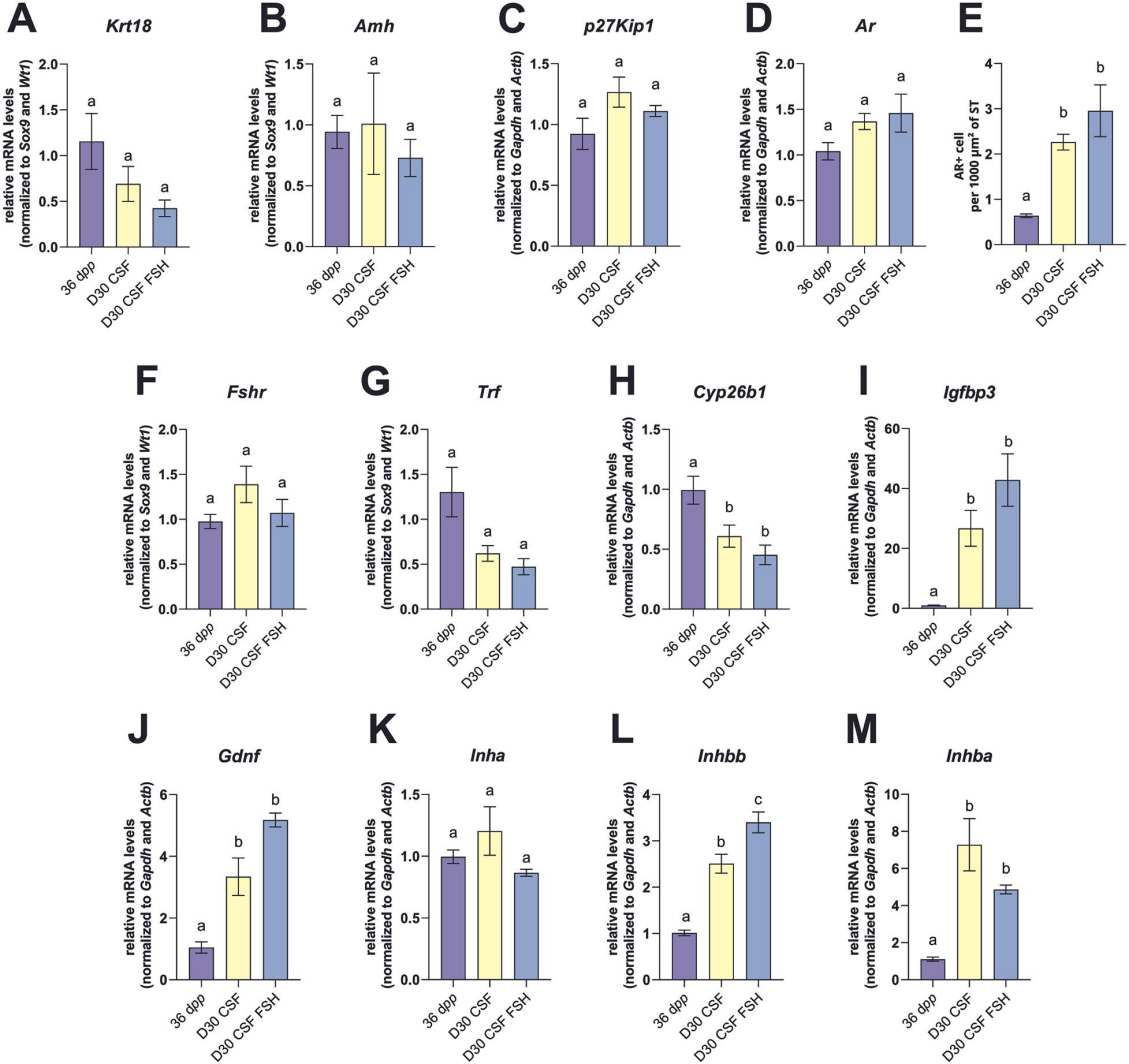
Effect of FSH on Sertoli cell maturation and functionality after 30 days of organotypic culture. **(A,B)** Relative mRNA levels of *Krt18* and *Amh* (immature Sertoli cell markers) normalized to *Sox9* and *Wt1* at 36 d*pp* and in 30-day cultured CSF tissues with or without 5 ng/mL of FSH. **(C,D)** Relative mRNA levels of *p27Kip1* and *Ar* (mature Sertoli cell markers) normalized to *Gapdh* and *Actb* at 36 d*pp* and D30 ± FSH. **(E)** Number of AR^+^ cells per 1,000 µm² of seminiferous tubule (ST) at 36 d*pp* and D30 ± FSH. **(F–M)** Relative mRNA levels of *Fshr*, *Trf*, *Cyp26b1, Igfbp3, Gdnf*, *Inha, Inhbb* and *Inhba* (normalized to *Sox9* and *Wt1* or to *Gapdh* and *Actb*). Data are presented as means ± SEM with *n* = 4–8 biological replicates for each group. Different letters denote significant differences between groups (*P* *<* 0.05). Identical letters between groups indicate that there is no significant difference between these groups (*P* ≥ 0.05). CSF, controlled slow freezing; D, days of culture; d*pp*, days *postpartum*; ST, seminiferous tubule.

The addition of FSH had no impact on the number of Sertoli cells per 1,000 µm² of ST, the percentage of Sertoli cells per ST and the germ to Sertoli cell ratio, which remained significantly different from *in vivo* controls (Figures 4C–E). In addition, no significant effect of FSH on *Sox9* and *Wt1* transcript levels was also observed at D30 (Figures 4F,G). Additionally, the expression of markers of Sertoli cell maturity was not significantly different in cultures supplemented or not with FSH (Figres 5A–E). Furthermore, the addition of 5 ng/mL FSH to the culture medium did not change *Inha*, *Inhba*, *Fshr*, *Trf*, *Igfbp3*, *Gdnf* transcript levels (Figures 5F–K, M). Only *Inhbb* mRNA levels were significantly increased in FSH supplemented cultures compared to unsupplemented ones (Figure 5L). *Inha*, *Fshr* and *Trf* mRNA levels were similar in cultured explants with or without FSH and in *in vivo* tissue controls (Figures 5F,G, K). In contrast, *Cyp26b1* transcript levels were lower in organotypic cultures than *in vivo* (Figure 5H) while *Inhba*, *Inhbb*, *Gdnf* and *Igfbp3* mRNA levels were higher in cultured explants than at 36 d*pp* (Figures 5I,J, L–M).

## Discussion

The low efficiency of *in vitro* spermatogenesis in cultures of frozen-thawed mouse prepubertal testicular tissues (2, 3) remains a major obstacle for the clinical application of this approach as a fertility restoration option for sterile pediatric cancer survivors. The question of whether Sertoli cells can progress toward maturity and acquire functional features in this organotypic culture system has received little attention to date. In the present study, the analysis of Sertoli cell maturity markers, combined with the measurement of intratesticular inhibin B levels in tissue cultures, supports the view that Sertoli cells acquire several maturation-associated features during culture and retain at least partial functional capacity. However, these findings should be interpreted cautiously, as they do not demonstrate complete or unequivocal Sertoli cell maturation under the present *in vitro* conditions.

The number of Sertoli cells is crucial for spermatogenesis, as it determines the number of germ cells per testis and sperm production (12). We first questioned whether the low sperm yield obtained in organotypic cultures (2, 3) could be attributed to a reduced number of Sertoli cells. We observed an increase in the number of Sertoli cells per 1,000 µm² of testicular tissue, undoubtedly due to the smaller size of seminiferous tubules in culture, which exhibited lower overall cell density and, most notably, a significant reduction in germ cells. This led to a higher Sertoli cell density in much smaller seminiferous tubules. These findings are consistent with the cell enrichment and germ-to-Sertoli cell ratio observed in the same culture system with frozen-thawed mouse prepubertal testicular tissues (5, 41).

In the present study, we further found that the expression levels of immature Sertoli cell markers (*Amh*, *Krt18*) and markers associated with a more advanced Sertoli cell state (*p27Kip1*, *Ar*) were broadly similar between *in vitro* cultured explants and *in vivo* tissue controls. An additional point requiring cautious interpretation concerns AMH detection. Although AMH was detectable in tissue homogenates, it remained undetectable in the culture medium. This discrepancy does not necessarily indicate a lack of AMH production by Sertoli cells *in vitro*, since the protein was measurable within the cultured tissue. Rather, several non-exclusive explanations may account for the lack of detection in the medium, including (*i*) low levels of AMH secretion, (*ii*) dilution effects related to culture medium volume and medium changes, (*iii*) the timing of medium collection relative to secretion kinetics, (*iv*) possible limited AMH stability under *in vitro* culture conditions, and (*v*) the possibility that part of the AMH produced is retained within the explant rather than being efficiently released into the surrounding medium. As these possibilities were not directly investigated here, the absence of detectable AMH in the medium should be interpreted cautiously.

Our data revealed that the number of AR-positive Sertoli cells per 1,000 µm² of seminiferous tubules was higher at D30 than at 36 d*pp*, likely due to the increased Sertoli cell density in seminiferous tubules at this organotypic culture time point. Nevertheless, the detection of AR in Sertoli cells should not be overinterpreted, as AR expression alone does not demonstrate restoration of normal AR-dependent Sertoli cell function. Indeed, the Sertoli cell-selective AR knockout (SCARKO) mouse model demonstrated that AR expression in Sertoli cells is not required to achieve a normal Sertoli cell number (46). Furthermore, despite AR expression, AR-dependent signaling may remain altered in Sertoli cells in organotypic cultures, as we previously showed that the expression of the AR-dependent Sertoli gene *Rhox5* was decreased at D30 compared with 36 d*pp* (5, 38). Taken together, these findings are consistent with partial Sertoli cell maturation rather than fully established Sertoli cell functionality. Thus, the higher density of AR-positive Sertoli cells in cultured explants should be interpreted as evidence of AR expression, but not as proof of fully restored androgen responsiveness.

Here, we demonstrated that the number of Sertoli cells per 1,000 µm² of seminiferous tubule was not significantly different between cultures supplemented or not with 5 ng/mL of FSH from D10. Excess estrogen and dysregulation of androgen and estrogen signaling in organotypic cultures (5) could potentially account for this increase in Sertoli cell numbers. Moreover, cultured Sertoli cells expressed the genes encoding the FSH receptor and transferrin at levels comparable to those observed *in vivo*, which is consistent with the acquisition of some mature features, although not necessarily full maturation.

It has been shown Sertoli cell maturation during puberty involves loss of their proliferative capacity, changes in gene and protein expression as well as the formation of the BTB (47). We previously reported that a functional BTB appears to be established and maintained in cultures of frozen-thawed prepubertal mouse testicular tissues, although *Cldn3* expression was decreased (38). This decrease in *Cldn3* expression observed in 30-day organotypic cultures, corresponding to 36 d*pp*, closely resembles the phenotype observed in 35 d*pp* and adult SCARKO mice (48), which have a Sertoli cell-specific deletion of the gene encoding the androgen receptor.

Differences in transcript levels were observed between *in vitro* explants and *in vivo* control matured tissues. For example, a decrease in the transcript levels of *Cyp26b1*, encoding the a*t*RA-degrading enzyme, was observed as early as 16 days of culture. The mechanisms regulating *Cyp26b1* gene expression are poorly understood; however, an autocrine negative feedback of a*t*RA has been suggested (49). The early decrease in *Cyp26b1* mRNA levels could therefore be due to the addition of Rol, a precursor of a*t*RA, into the culture medium from D2 to stimulate the entry into meiosis.

Additionally, a 25- to 27-fold increase in *Igfbp3* mRNA levels was observed in *in vitro* matured explants compared to control testes. These results are consistent with previous transcriptomic analysis of cultured fresh and frozen-thawed mouse prepubertal testicular tissues with the detection of *Igfbp3* as the most deregulated gene (overexpressed) at D16 and D30, in comparison to their respective *in vivo* controls at 22 d*pp* and 36 d*pp* (37) In addition, it has been shown in rat Leydig cells that IGFBP3 did not affect hCG-induced testosterone synthesis in the absence of IGF1 (50). Indeed, we previously reported an increase in testosterone production in response to hCG in organotypic cultures despite *Igfbp3* overexpression (5). Nevertheless, excess IGFBP3 could be detrimental to spermatogenesis progression, as it is known to inhibit IGF1 action in Sertoli cells (18) and to promote germ cell apoptosis (51).

We previously demonstrated that supplementation of organotypic culture media with 500 IU/L of FSH (around 35 ng/mL) together with 50 IU/L hCG from D7 onwards resulted in a slight increase in the production of spermatozoa (2). To better reproduce *in vivo* conditions, we added FSH at a more physiological dose (5 ng/mL) from D10 instead of D7. Indeed, immature Sertoli cells proliferate up to 16–17 d*pp*, corresponding to D10-D11 of culture (13, 52). However, supplementation with 5 ng/mL of FSH from D10 had little impact on the culture of frozen-thawed prepubertal mouse testicular tissues. Indeed, previous studies have shown that stimulation of neonatal mouse testicular cells on decellularized testicular matrix with gonadotrophins (5 IU/L FSH and hCG) did not further increase inhibin B and testosterone secretion by Sertoli and Leydig cells, respectively (53, 54). In addition, although FSH is known to be a potent and rapid inhibitor of *Igfbp3* mRNA expression (18), the addition of 5 ng/mL of FSH failed to reduce *Igfbp3* expression. Moreover, the addition of FSH had no impact on mRNA levels of *Amh*, *Inhba*, *Trf* or *Gdnf*, known to be regulated by FSH. Because FSH regulates signaling pathways interacting with the active metabolite of Rol, A*t*RA, to commit spermatogonia to the differentiation pathway and meiosis (54), the attenuated FSH responsiveness together with altered AR/A*t*RA crosstalk observed in our cultures may contribute to the incomplete progression of spermatogenesis despite evidence that Sertoli cells acquire only partial maturation-associated features *in vitro*. However, A*t*RA inhibits FSH transduction pathways in Sertoli cells, by blocking the production of cyclic AMP. In turn; FSH suppresses the a*t*RA-induced nuclear localization, transcriptional transactivation, and protein expression of the retinoic acid receptor alpha (RAR*α*) (55, 56). Therefore, it seems appropriate not to supplement FSH at the same time as Rol in the culture medium (2).

A more in-depth understanding of all the molecular mechanisms limiting the progression of *in vitro* spermatogenesis is required to determine the necessary supplementations for optimizing this organotypic culture procedure. Developing an effective model of *in vitro* spermatogenesis could not only advance the field of fertility restoration but also have broader implications. This model could be invaluable for studying the onset of puberty and evaluating the effects of cancer treatments, drugs and environmental factors, such as endocrine disruptors, on the developing testis.

In conclusion, the present study supports that Sertoli cells in cultures of frozen-thawed prepubertal mouse testicular tissues acquire several maturation-associated and functional features, but remain only partially matured under the present conditions. In particular, the limited effect of FSH supplementation, the persistent alterations in *Igfbp3* and *Cyp26b1* expression, and the remaining uncertainty regarding AR-dependent function indicate that the *in vitro* system does not yet fully recapitulate physiological Sertoli cell maturation. These limitations, together with impaired Leydig cell differentiation, may contribute to the low efficiency of *in vitro* spermatogenesis.

## Data Availability

The raw data supporting the conclusions of this article will be made available by the authors, without undue reservation.

## Footnotes

^1^ https://www.R-project.org/

^2^ http://www.rstudio.com/

## Supplementary material

Supplementary Figure S1 Organotypic Culture Design. In this study, prepubertal testicular tissues from 6 d*pp* mice were collected and stored in liquid nitrogen following controlled slow freezing (CSF). After thawing, testicular fragments were cultured at a gas-liquid interphase, under 5% CO_2_ at 34°C, during 16 days (D16) or 30 days (D30). Testicular explants were cultured with BM (α-MEM without phenol red, 10% KSR, 1 µM retinol every 8 days, antibiotics), which was supplemented or not with 5 ng/mL FSH from D10. Testes from mice aged 22 and 36 d*pp* were used as the *in vivo* controls for D16 and D30, respectively. In the scheme representing the organotypic culture timeline, arrows represent the times at which culture media were collected (D0, D2, D6, D10, D14, D18, D22, D26, and D30). BM, basal medium; CSF, controlled slow freezing; D, days of culture; d*pp*, days *postpartum*; FSH, follicle stimulating hormone; KSR, KnockOut Serum replacement; PR-, medium without phenol red; Rol, retinol.

Supplementary Figure S2 Typical representation of the CREM testicular marker. Representative images of CREM expression at 36 d*pp* and in 30-day cultured CSF tissues without or with 1, 5 or 10 ng/mL of FSH. Testicular tissue sections were counterstained with hematoxylin. Scale: 50 µm. Photomicrographs at a 5× additional digital magnification are shown. Yellow arrowheads indicate round spermatid cell. CSF, controlled slow freezing; D, days of culture; d*pp*, days *postpartum*.

Supplementary Table S1 List of PCR primers used in this study.
