# Supplementary figures and images for: Maturation-associated changes in Sertoli cells following in vitro culture of frozen-thawed prepubertal mouse testicular tissue
Source: Front Reprod Health. 2026 Apr 23;8:1835316. doi: 10.3389/frph.2026.1835316 (PMC13227114; doi:10.3389/frph.2026.1835316)

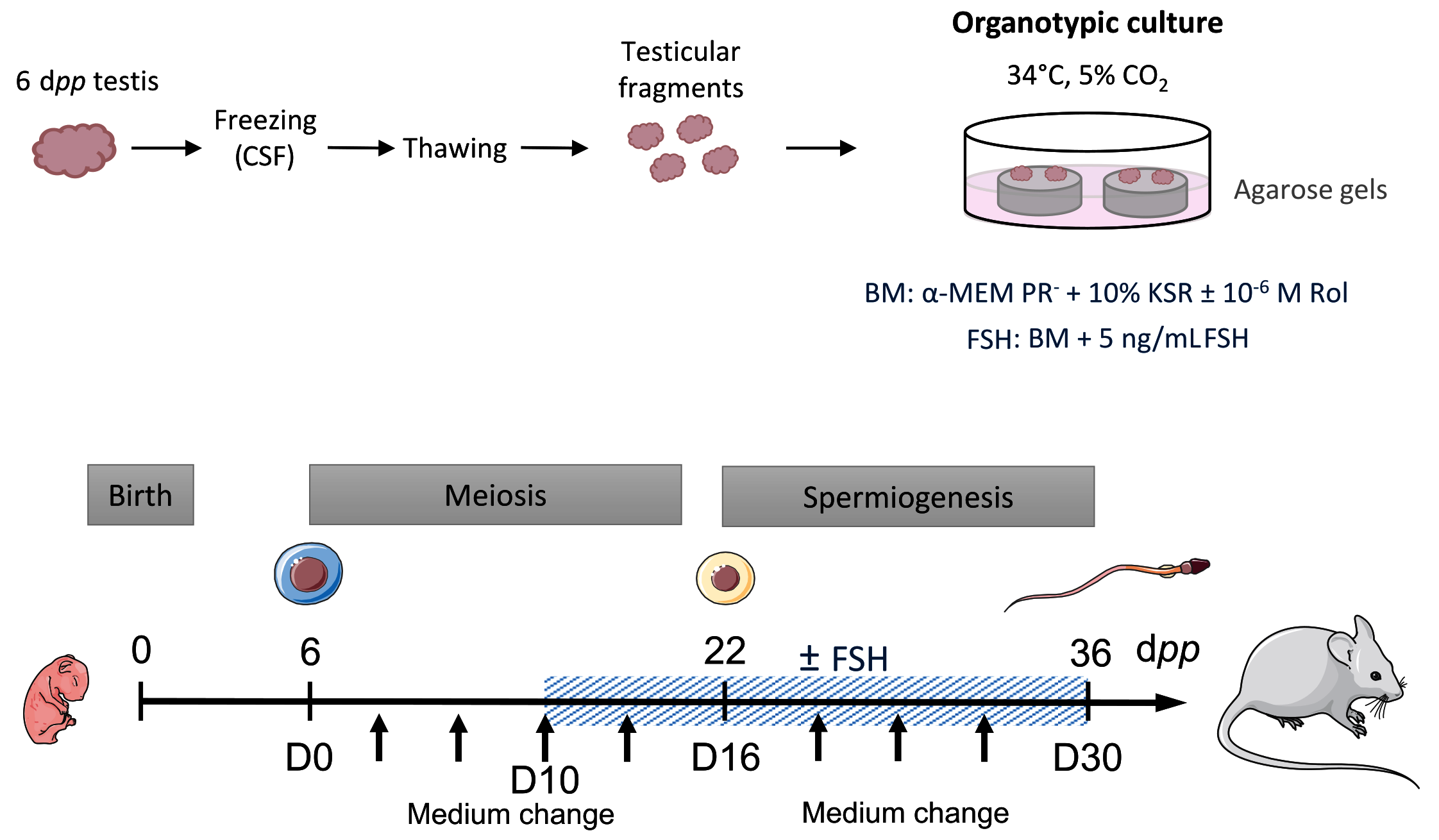

Supplement: Supplementary Figure S1 — Organotypic Culture Design. In this study, prepubertal testicular tissues from 6 dpp mice were collected and stored in liquid nitrogen following controlled slow freezing (CSF). After thawing, testicular fragments were cultured at a gas-liquid interphase, under 5% CO2 at 34°C, during 16 days (D16) or 30 days (D30). Testicular explants were cultured with BM (α-MEM without phenol red, 10% KSR, 1 µM retinol every 8 days, antibiotics), which was supplemented or not with 5 ng/mL FSH from D10. Testes from mice aged 22 and 36 dpp were used as the in vivo controls for D16 and D30, respectively. In the scheme representing the organotypic culture timeline, arrows represent the times at which culture media were collected (D0, D2, D6, D10, D14, D18, D22, D26, and D30). BM, basal medium; CSF, controlled slow freezing; D, days of culture; dpp, days postpartum; FSH, follicle stimulating hormone; KSR, KnockOut Serum replacement; PR-, medium without phenol red; Rol, retinol. [file Image1.tif]

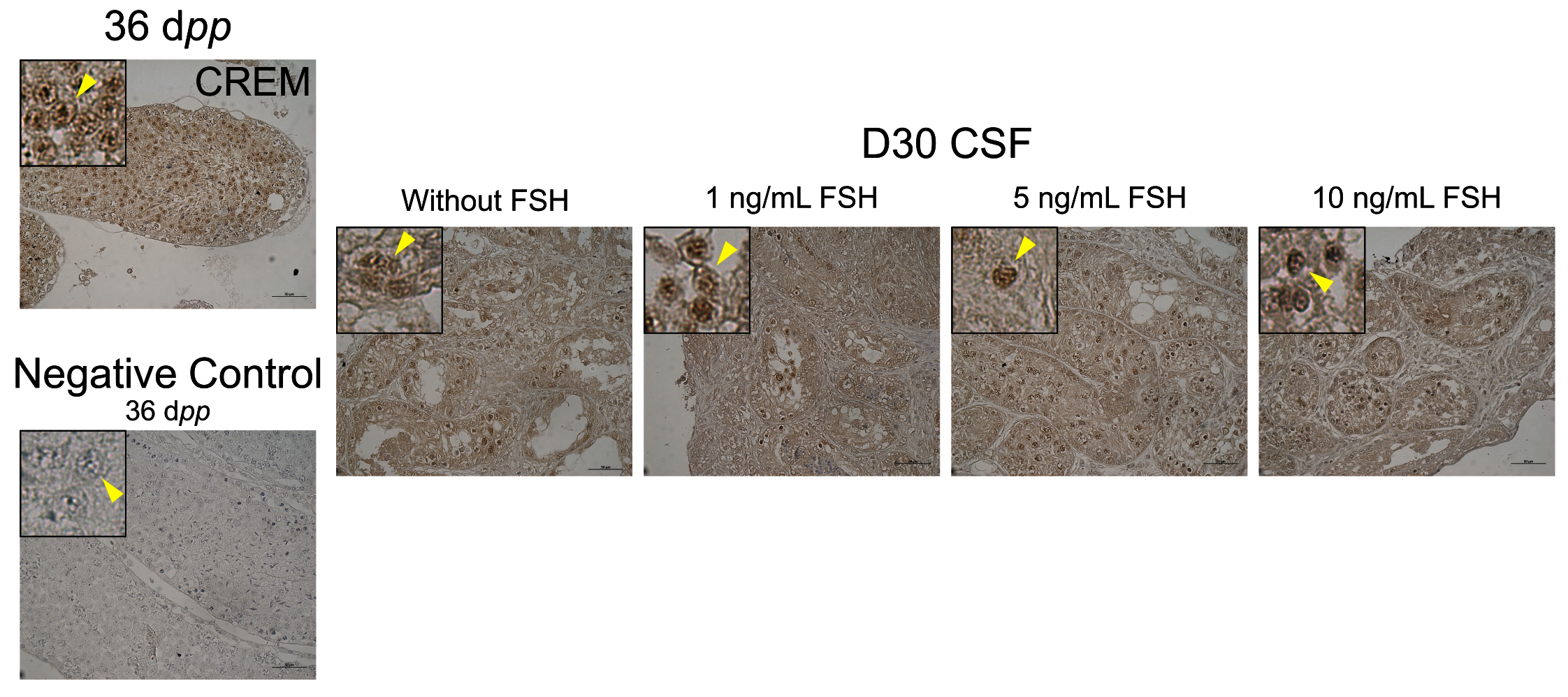

Supplement: Supplementary Figure S2 — Typical representation of the CREM testicular marker. Representative images of CREM expression at 36 dpp and in 30-day cultured CSF tissues without or with 1, 5 or 10 ng/mL of FSH. Testicular tissue sections were counterstained with hematoxylin. Scale: 50 µm. Photomicrographs at a 5× additional digital magnification are shown. Yellow arrowheads indicate round spermatid cell. CSF, controlled slow freezing; D, days of culture; dpp, days postpartum. [file Image2.tif]
